# Neuronal nitric oxide synthase required for erythropoietin modulation of heart function in mice

**DOI:** 10.3389/fphys.2024.1338476

**Published:** 2024-04-02

**Authors:** Jeeyoung Lee, Heather M. Rogers, Danielle A. Springer, Constance T. Noguchi

**Affiliations:** ^1^ Molecular Medicine Branch, National Institute of Diabetes and Digestive and Kidney Diseases, National Institutes of Health, Bethesda, MD, United States; ^2^ Murine Phenotyping Core, National Heart, Lung, and Blood Institute, National Institutes of Health, Bethesda, MD, United States

**Keywords:** erythropoietin, neuronal nitric oxide synthase, heart function, heart failure-associated genes, nNOS-knockout mice, hematocrit

## Abstract

**Introduction:** Erythropoietin (EPO) acts primarily in regulating red blood cell production mediated by high EPO receptor (EPOR) expression in erythroid progenitor cells. EPO activity in non-erythroid tissue is evident in mice with EPOR restricted to erythroid tissues (ΔEPORE) that become obese, glucose-intolerant, and insulin-resistant. In animal models, nitric oxide synthase (NOS) contributes to EPO activities including erythropoiesis, neuroprotection, and cardioprotection against ischemia-reperfusion injury. However, we found that extended EPO treatment to increase hematocrit compromised heart function, while the loss of neuronal NOS (nNOS) was protective against the deleterious activity of EPO to promote heart failure.

**Methods:** Wild-type (WT) mice, ΔEPORE mice, and *nNOS*-knockout mice (*nNOS*−/−) were placed on a high-fat diet to match the ΔEPORE obese phenotype and were treated with EPO for 3 weeks. Hematocrit and metabolic response to EPO treatment were monitored. Cardiac function was assessed by echocardiography and ultrasonography.

**Results:** ΔEPORE mice showed a decrease in the left ventricular outflow tract (LVOT) peak velocity, ejection fraction, and fractional shortening, showing that endogenous non-erythroid EPO response is protective for heart function. EPO treatment increased hematocrit in all mice and decreased fat mass in male WT, demonstrating that EPO regulation of fat mass requires non-erythroid EPOR. EPO treatment also compromised heart function in WT mice, and decreased the pulmonary artery peak velocity (PA peak velocity), LVOT peak velocity, ejection fraction, and fractional shortening, but it had minimal effect in further reducing the heart function in ΔEPORE mice, indicating that the adverse effect of EPO on heart function is not related to EPO-stimulated erythropoiesis. ΔEPORE mice had increased expression of heart failure-associated genes, hypertrophic cardiomyopathy-related genes, and sarcomeric genes that were also elevated with EPO treatment in WT mice. Male and female *nNOS*−/− mice were protected against diet-induced obesity. EPO treatment in *nNOS−/−* mice increased the hematocrit that tended to be lower than WT mice and decreased the PA peak velocity but did not affect the LVOT peak velocity, ejection fraction, and fractional shortening, suggesting that nNOS is required for the adverse effect of EPO treatment on heart function in WT mice. EPO treatment did not change expression of heart failure-associated gene expression in *nNOS−/−* mice.

**Discussion:** Endogenous EPO has a protective effect on heart function. With EPO administration, in contrast to the protective effect to the cardiac injury of acute EPO treatment, extended EPO treatment to increase hematocrit in WT mice adversely affected the heart function with a corresponding increase in expression of heart failure-associated genes. This EPO activity was independent of EPO-stimulated erythropoiesis and required EPOR in non-erythroid tissue and nNOS activity, while *nNOS*−/− mice were protected from the EPO-associated adverse effect on heart function. These data provide evidence that nNOS contributes to the negative impact on the heart function of high-dose EPO treatment for anemia.

## 1 Introduction

Erythropoietin (EPO), a hormone synthesized in the kidney, regulates red blood cell production (Bhoopalan et al., 2020). Anemia and chronic kidney disease are independently associated with an increased risk of cardiovascular events and death among patients with heart failure (McClellan et al., 2002). Initial studies showed that in anemic patients with chronic kidney disease on dialysis, EPO treatment was administered three times weekly after dialysis dose dependently increased hematocrit, up to 10 percent at 3 weeks, and reduced the transfusion requirement (Eschbach et al., 1987). Recombinant human EPO used to treat anemia in chronic kidney disease due to low EPO production helped improve the cardiac function (Silverberg et al., 2002). Animal studies provide evidence that EPO has protective activity in non-hematopoietic tissues including the vascular endothelium, brain, skeletal muscle, and adipose tissue (Suresh et al., 2019b). A single injection of EPO that does not alter hematocrit was shown to have protective activity in animal models of heart ischemia-reperfusion injury (Parsa et al., 2003; Cai and Semenza, 2004). Animal models of EPO cardioprotective activity include mice, rats, rabbits, dogs, pigs, and sheep (Parsa et al., 2003; Cai and Semenza, 2004; Hirata et al., 2005; Schneider et al., 2007; Chua et al., 2011; Olea et al. 2013). However, in human studies, to assess the benefit on the cardiovascular health of long-term high-dose EPO treatment for anemia in chronic kidney disease, no benefit to heart health was found. Using high-dose EPO for the complete correction of anemia or to achieve a high hemoglobin level, no reduction in the risk of cardiovascular events was observed; rather, there was an increased risk of serious cardiovascular events and mortality (Drueke et al., 2006; Singh et al., 2006; McCullough et al., 2013). Transgenic mice expressing high human EPO reach hematocrits of 80% and exhibit normal blood pressure and heart rate attributed to increased nitric oxide (NO) production (Ruschitzka et al., 2000). This is consistent with the EPO-stimulated endothelial response and increased endothelial NO synthase (eNOS) activation (Anagnostou et al., 1994; Beleslin-Cokic et al., 2004). Administration of the NO synthase (NOS) inhibitor to these high-EPO transgenic mice resulted in death within 52 h accompanied by acute left dilation of the heart, vasoconstriction of muscular arteries of the systemic circulation, and severe pulmonary congestion (Ruschitzka et al., 2000). However, in contrast to the protective effect of single-dose EPO treatment in mouse models of ischemia-reperfusion injury (Burger et al., 2006; Teng et al., 2011a), these transgenic mice with chronic exposure to high EPO exhibited cardiac dysfunction, ventricular dilation, reduced exercise performance, and reduced life span (Wagner et al., 2001), suggesting adverse impact on heart function with chronic high-dose EPO. Here, we assessed the effect on heart health in mice treated with high-dose EPO for 3 weeks to increase hematocrit and the potential protective effect of NOS activity on the EPO cardiovascular activity.

## 2 Materials and methods

### 2.1 Animals

Animal procedures were approved by the National Institute of Diabetes and Digestive and Kidney Diseases Animal Care and Use Committee (ASP K085-MMB-21) and carried out in accordance with the National Institutes of Health Guidelines for the Care and Use of Laboratory Animals. Mice were housed in a specific pathogen-free environment under a controlled temperature (23°C) and a 12 h light/dark cycle with free access to food (NIH-31, 14% kcal/fat, 3.0 kcal/g, Teklad Diets) and water.

**FIGURE 1:**
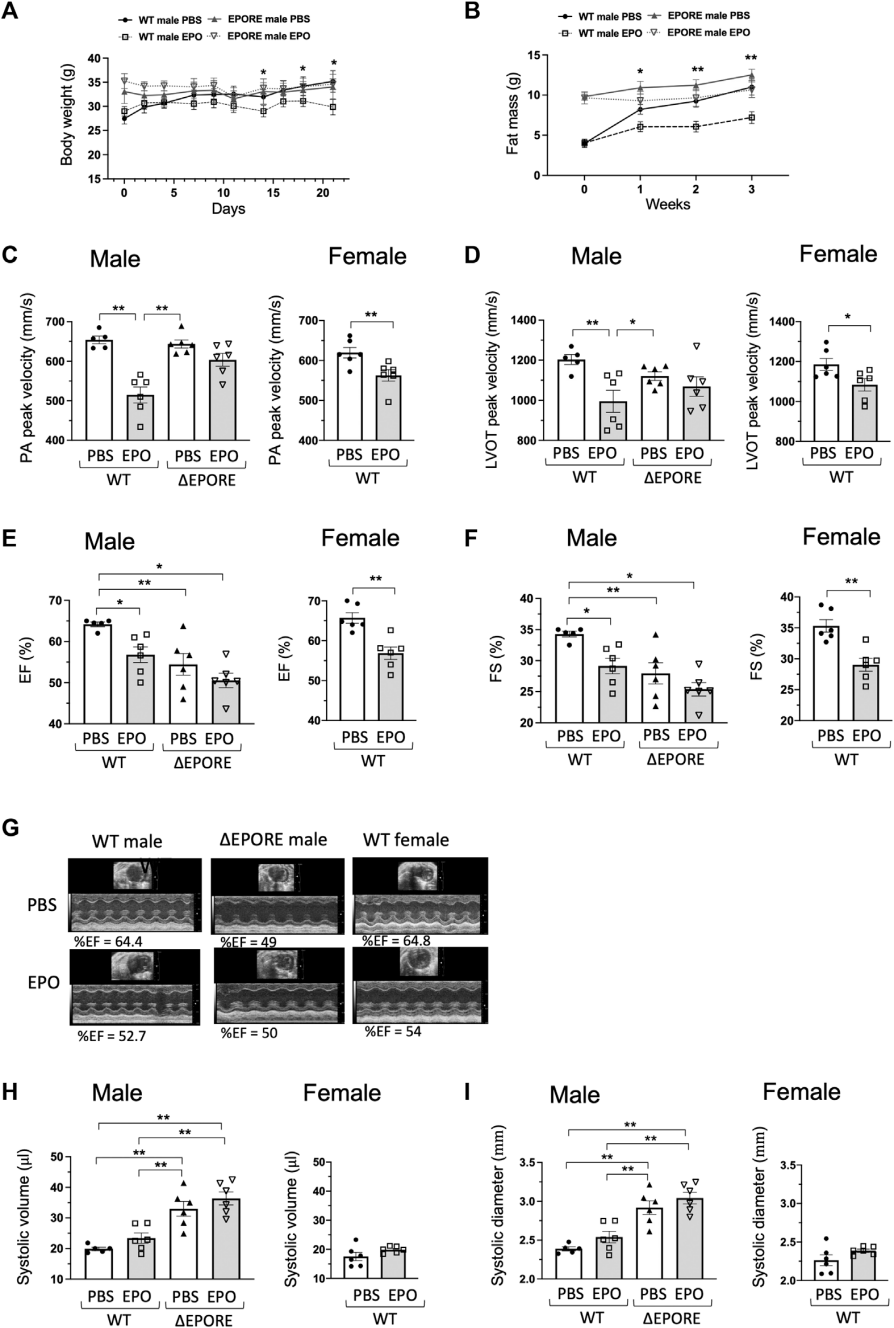
EPO modulation of heart function. **(A,B)** EPO treatment with a high-fat diet decreased body weight **(A)** and fat mass accumulation **(C)** in WT male mice but not in ∆EPORE male mice. **(C,D)** EPO treatment with a high-fat diet significantly reduced the PA peak velocity **(C)** and LVOT peak velocity **(D)** of flow in WT male and female mice but not in ∆ EPORE male mice. **(E,F)**. EPO significantly reduced the ejection fraction (EF) **(E)** and fractional shortening (FS) **(F)** in WT male and female mice but not in ΔEPORE male mice that have the lowest level of ejection fraction **(E)** and fractional shortening **(F)**. **(G)** Representative echocardiographic images. **(H,I)** EPO did not change the systolic volume **(H)** and systolic diameter **(I)** in all groups. * <0.05; ** <0.01.

All the mice had a C57BL/6 background. EPO acts by binding to the cell surface EPO receptor (EPOR) that is expressed at the highest level on erythroid progenitor cells. Mice with targeted deletion of EPOR (*Epor*−/−) die of severe anemia during embryonic development (Wu et al., 1999; Yu et al., 2001). ΔEPORE mice that are EPOR−/− mice rescued by an erythroid-restricted *EPOR* transgene (GATA-1 locus hematopoietic regulatory domain-driving mouse *EPOR* cDNA) (Suzuki et al., 2002) were used as models for the lack of EPOR signaling in non-erythroid cells. ΔEPORE mice exhibit appropriate EPO-stimulated erythropoietic response (Supplementary Figure S1A) and have an obese phenotype with an increased body weight and body fat mass and develop glucose intolerance and insulin resistance (Figures 1A, B; Supplementary Figures S1B–D) (Teng et al., 2011b). For an obese phenotype, we follow previous observations for obese mice on a high-fat diet compared with standard chow that were reported to have a percentage body fat of 36.8% (±12.3%) vs. 15.8 (±5.8%), respectively (Kaiyala et al., 2010). Heterozygous neuronal NOS (nNOS)-knockout (*nNOS+/−*) mice (Jackson Laboratory, Bar Harbor, ME, United States) were mated with each other to produce *nNOS*−/−mice.

At 4 months of age, all mice were fed a high-fat diet (60 kcal% fat, 34.9% crude fat, 5.24 kcal/kg, Research Diets Inc.) for 3 weeks to match the ΔEPORE obese phenotype [high-fat diet feeding does not exacerbate heart failure (Brainard et al., 2013)] and treated with or without EPO (3,000 units/kg three times/week of recombinant human EPO, Epogen, Amgen, Thousand Oaks, CA, United States). Body composition was measured using a three-in-one EchoMRI (Echo Medical Systems, Houston, TX, United States). Hematocrit was measured manually before and every week after EPO administration.

### 2.2 Glucose tolerance test and insulin tolerance tests

Mice were fasted overnight and were then injected intraperitoneally with glucose (2.0 g kg^−1^ body weight) or insulin (humulin R, 0.75 mU kg^−1^). Blood glucose levels were measured before (0 min) and up to 120 min (0, 15, 30, 60, and 120 min) after the injection by withdrawal of 3 μL blood from the tail vein. The glucose level was determined using the Elite Glucometer (Bayer, Whippany, NJ, United States), following the procedure of Chen et al. (2005).

### 2.3 Two-dimensional and M-mode echocardiograph

Transthoracic echocardiography was carried out using a high-frequency, high-resolution ultrasound system (Vevo2100, FUJIFILM VisualSonics Inc., Toronto, Canada) and a 40-MHz transducer probe (VisualSonics, MS-400). Mice were lightly anesthetized with 1%–2% isoflurane to achieve a target heart rate of >400 beats per minute and positioned over a heated platform with EKG, respiration, and body temperature monitoring. Two-dimensional images were obtained for multiple views or the heart, and M-mode images from the short axis mid-papillary view of the left ventricle (LV) were used for measurements of the systolic and diastolic posterior and anterior wall thicknesses, end systolic, and end diastolic internal LV chamber dimensions (LVIDs and LVIDd), and the LV functional values of fractional shortening (FS) and ejection fraction (%EF) were calculated within the systems analysis software program.

### 2.4 Pulsed-wave (PW) and color Doppler

Color and PW Doppler ultrasonography was also performed to assess the pulmonary artery and aortic blood flow. Color flow profiles were obtained for the left ventricular outflow tract (LVOT) from a suprasternal view. For generating the pulmonary artery color flow profiles, the probe was moved slightly cranial and leftward from the parasternal long axis view until the pulmonary artery was observed crossing over the aorta. The color flow images served as a guide for placement of the PW Doppler sample volume to obtain peak aortic and pulmonary artery velocities.

### 2.5 Quantitative real-time RT-PCR

The left bottom of the heart from mice was used for total RNA extraction, and 1 μg of total RNA was reverse-transcribed (Invitrogen, Carlsbad, CA, United States). Quantitative real-time RT-PCR analyses were carried out using gene-specific primers (Supplementary Table S1) and fluorescently labeled SYBR Green dye (Roche Applied Science, IN, United States) in a 7900 Sequence Detector (PE Applied Biosystems, Foster City, CA, United States). Relative mRNA quantification was calculated by the delta–delta Ct method, and Ct values were normalized with *RPL13a* as an internal control.

### 2.6 Western blotting

Heart tissues were homogenized and lysed in denaturing radioimmunoprecipitation (RIPA) assay buffer supplemented with protease and phosphatase inhibitors (Roche). Proteins were electrophoretically separated with 4%–20% Tris-glycine SDS/PAGE gels, transferred to nitrocellulose membranes using an XCell SureLock Mini-Cell system (Invitrogen), and visualized using protein-specific antibodies. The following antibodies were used: pERK (Cell Signaling Technology; #5726; 1:1000, Danvers, MA, United States), ERK (Cell Signaling Technology; #4695; 1:1000), Nox4 (Novus Biologicals; NB110-58849; 1:1000, Littleton, CO, United States), iNOS (Abcam; Ab3523; 1:1000, Boston, MA), pAkt (Cell Signaling Technology; #9271; 1:1000), Akt (Cell Signaling Technology; #9272; 1:1000), and GAPDH (Cell Signaling Technology; #97166; 1:1000). Quantitative analysis was performed by measuring the integrated density with an NIH ImageJ system and normalized with GAPDH.

### 2.7 Statistical analysis

The data are expressed as mean ± SEM. Student’s two-tailed non-paired *t*-test was used, and *p* values of <0.05 were regarded statistically significant. One-way analysis of variance (ANOVA) adjusted by Fisher LSD was used for multiple comparisons.

## 3 Results

### 3.1 ΔEPORE mice with EPOR restricted to erythroid tissue

All mice at 4 months of age were fed high-fat diet with and without EPO treatment to match the ΔEPORE obese phenotype (Figures 1A, B). EPO treatment for 3 weeks showed comparable erythropoietic response and an increase in hematocrit in male and female WT and ΔEPORE mice (Supplementary Figure S1A). To determine the influence of endogenous EPO activity on heart function, we used echocardiography to assess the cardiac function in WT male and ΔEPORE male mice with EPOR restricted to erythroid tissue. ΔEPORE male mice showed a similar level of PA peak velocity compared to WT male mice (Figure 1C). Without EPO treatment, ΔEPORE male mice showed a decreased LVOT peak velocity, ejection fraction, and fractional shortening compared to WT male mice (Figures 1D–G), suggesting that the endogenous EPO response of non-hematopoietic cells in WT mice protects the cardiac function. ΔEPORE mice also showed an increased systolic volume and systolic diameter compared to WT male mice, suggesting that in WT mice, endogenous EPO in non-hematopoietic tissues might play a role in protecting against hypertrophic cardiomyopathy (Figures 1H, I).

### 3.2 High-dose exogenous EPO can harm cardiac function

The impact on the heart function of EPO administration to increase hematocrit was determined by echocardiography to assess the cardiac function with EPO or PBS treatment in WT male, WT female, and ΔEPORE male mice. ΔEPORE male mice showed a similar level of PA peak velocity compared to WT male mice (Figure 1C). Although EPO treatment increased hematocrit similarly between WT and ΔEPORE mice (Supplementary Figure S1A), EPO treatment reduced the PA peak velocity of blood flow in male and female WT mice but did not affect the PA peak velocity in ΔEPORE male mice (Figure 1C). EPO treatment reduced the LVOT peak velocity, ejection fraction, and fractional shortening in WT male and female mice but had no effect in ΔEPORE mice (Figures 1D–G), indicating that high-dose exogenous EPO can harm the cardiac capability, that this response is mediated via *EPOR* expression in non-erythroid tissue and independent of change in hematocrit, and that high-dose EPO cannot modulate the cardiac function in mice that lack *EPOR* in non-hematopoietic cells. Although the systolic volume and systolic diameter were increased in ΔEPORE male mice compared to WT male mice, EPO treatment did not change the systolic volume and diameter in all mouse groups (Figures 1H, I).

### 3.3 High-dose EPO treatment regulated cardiac function-related genes

**FIGURE 2:**
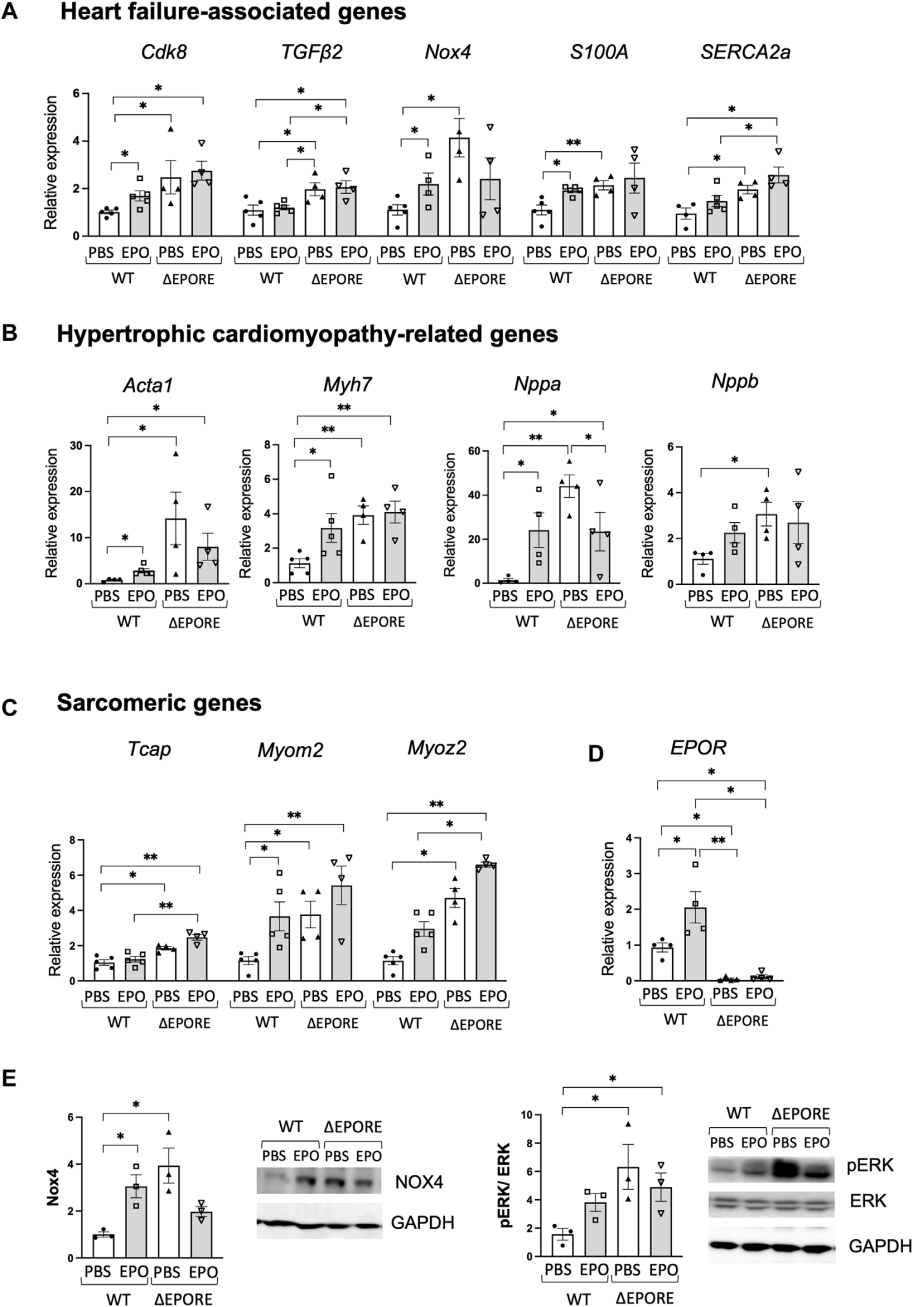
EPO regulation of heart failure-associated genes. **(A–C)** EPO enhanced expression of heart failure-associated genes **(A)**, hypertrophic cardiomyopathy-related genes **(B)**, and sarcomeric genes **(C)** in WT male mouse cardiac tissues, while ΔEPORE male mice have higher expression levels of heart failure-associated genes **(A)** and hypertrophic cardiomyopathy-related genes **(B)** and sarcomeric genes **(C)** compared to WT male mice (*n* = 4–5). **(D)** EPO increased *EPOR* gene expression in WT mouse cardiac tissues, and *EPOR* was not expressed in cardiac tissue of ΔEPORE mice (*n* = 4–5). **(E)** Western blotting for Nox4 (left), ERK and pERK (right), and GAPDH as control was carried out for cardiac tissue of WT and ΔEPORE mice treated with EPO and PBS. *n* = 3, * <0.05, ** <0.01.

EPOR expression in the cardiovascular system including endothelial cells and cardiomyocytes provides for EPO response. The cardioprotective effect of single bolus EPO administration after myocardial infarction observed in rats has been attributed to neovascularization and EPO-stimulated cardiomyocyte vascular endothelial growth factor production (van der Meer et al., 2005; Westenbrink et al., 2010). Here, we found that EPO treatment over 3 weeks to increase hematocrit adversely affected heart function and that this EPO modulation of heart function in WT mice was mediated by EPOR expression beyond erythroid tissue since EPO treatment had little effect on heart function in ΔEPORE mice (Figure 1). We examined how endogenous EPO activity contributed to the regulation of heart failure-associated gene expression by comparing expression in WT and ΔEPORE male mice. Among the genes examined were Cdk8, for which increased transgenic expression in mice promotes heart failure (Hall et al., 2017), TGFβ that exhibits elevated levels in myocardial infarction (Liu et al., 2017), and Nox4 that is a major source of reactive oxygen species (Gray et al., 2019). ΔEPORE mice showed increased expression of two-fold or more of heart failure-associated genes *Cdk8, TGFb2, Nox4, S100A,* and *SERCA2a* (Figure 2A).

Among hypertrophic cardiomyopathy-related genes, high phenotypic expression of Myh7 affects the myocardial mechanical function (de Frutos et al., 2022), Nppa expression reactivated in the ventricles is a marker of heart disease (Horsthuis et al., 2008), and elevated BNP (Nppb) is a hallmark of heart failure (Sangaralingham et al., 2023). ΔEPORE mice also expressed more than two-fold increased expression of hypertrophic cardiomyopathy-related genes, *Acta1, Myh7, Nppa,* and *Nppb* (Figure 2B). Sarcomere dysfunction contributes significantly to heart failure and includes, for example, genetic mutations in myomesin 2 (Myom2) located in the M line, middle of the sarcomere, and provides structural integrity, myozenin 2 (Myoz2) located in the Z line and regulates calcium-dependent signaling pathways, and possibly Tcap also in the Z-line (Sequeira et al., 2014; Martin et al., 2021). ΔEPORE male mice also exhibited increased expression of sacromeric genes, *Tcap, Myom2,* and *Myoz2* compared to WT male mice (Figure 2C). Upregulated expression of these genes was consistent with the cardiac dysfunction and hypertrophic cardiomyopathy phenotype in ΔEPORE male mice (Figure 1). The data suggest that endogenous EPO regulates cardiac dysfunction-related genes and sarcomeric genes that are increased with the loss of *EPOR* in non-erythroid cells including cardiac tissues in ΔEPORE mice (Figure 2).

High-dose EPO treatment increased *EPOR* in cardiac tissues in WT mice but not in ΔEPORE mice (Figure 2D). High-dose EPO also increased heart failure-associated genes such as *Cdk8, Nox4, S100A,* and *SERCA2a*, and hypertrophic cardiomyopathy-related genes such as *Acta1, Myh7, Nppa,* and *Nppb* in male WT mice but not in ΔEPORE male mice (Figures 2A, B). Examination by Western blotting showed Nox4 protein was increased in male ΔEPORE mice compared with male WT mice, and EPO treatment in WT male mice increased Nox4 protein, but EPO-treated ΔEPORE mice showed a trend of decreased Nox4 protein (Figure 2E, left). Western blotting demonstrated that changes in Nox4 protein corresponded to changes in gene expression. Extracellular signal-regulated kinase (ERK) contributes to cardiac function, can be stimulated by growth factors to promote proliferation or prevent apoptosis, or, conversely, contributes to cardiac hypertrophy and progression to heart failure (Gallo et al., 2019). We previously observed that in a mouse model of heart injury, acute EPO treatment increased ERK signaling and protected against ischemic-reperfusion injury in mice (Teng et al., 2011a). Here, we found that the cardiac dysfunction and hypertrophic cardiomyopathy phenotype in ΔEPORE male mice was accompanied by enhanced pERK compared to WT mice (Figure 2E, right). In addition, EPO treatment in WT male mice compromised the heart function and increased pERK, but EPO-treated ΔEPORE mice showed minimal changes in pERK (Figure 2E, right). This raises the possibility that pERK signaling plays a critical role for both endogenous and exogenous EPO responses in cardiac modulation.

### 3.4 EPO treatment changed glucose tolerance and hematocrit in *nNOS*−/− mice

**FIGURE 3:**
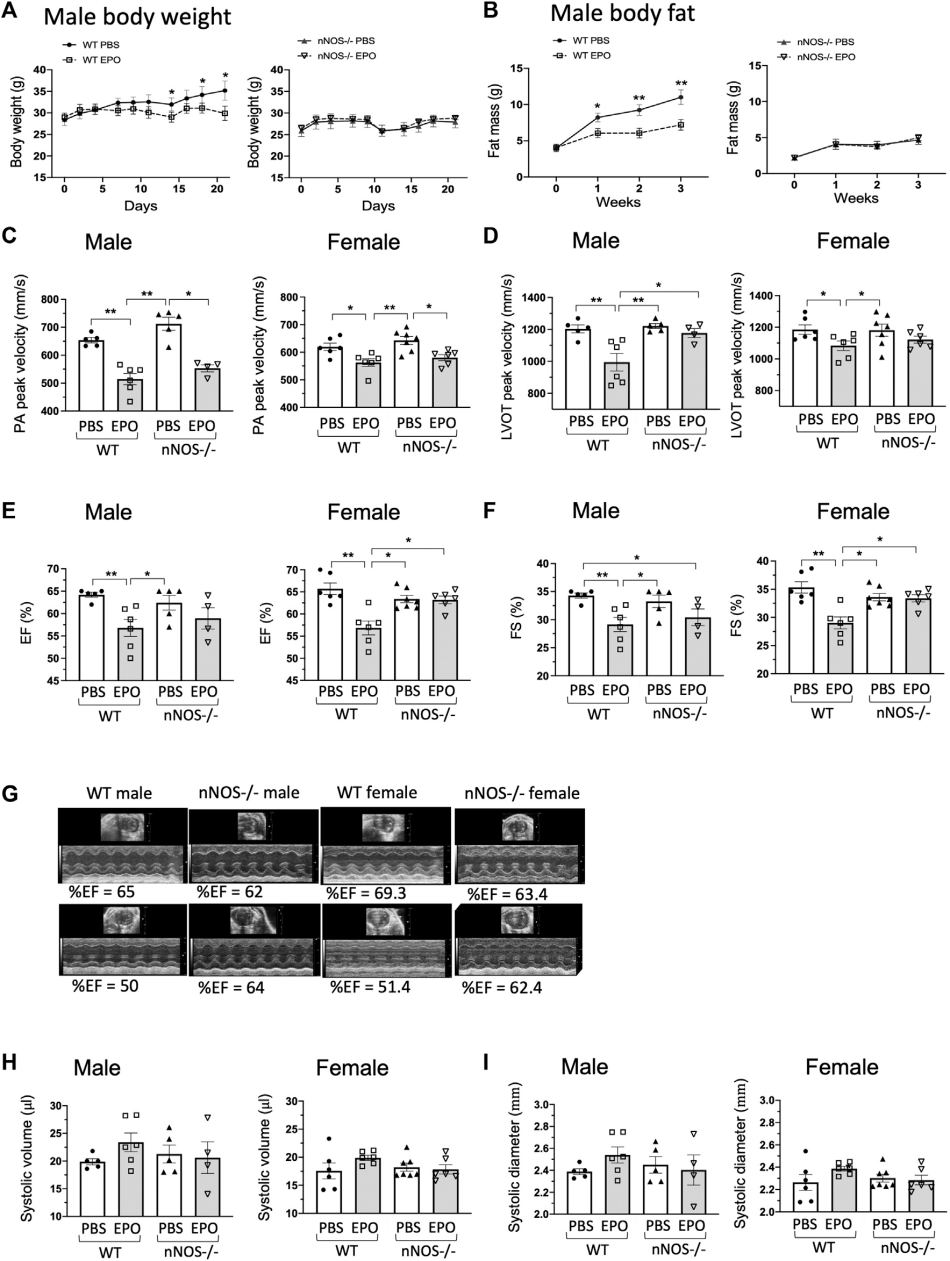
EPO did not change ejection fraction and fractional shortening of nNOS−/− mice **(A,B)**. EPO treatment with a high-fat diet decreased body weight **(A)** and fat mass accumulation **(B)** in WT male mice but not in *nNOS*−/− mice. *n* = 4–7, *, ** mean PBS vs. EPO in WT mice. **(C)** EPO reduced the PA peak velocity of flow in all groups. **(D)** Decreased LVOT peak velocity was shown in EPO-treated WT male and female mice but not in EPO-treated *nNOS*−/− male and female mice. **(E,F)** EPO reduced ejection fraction **(E)** and fractional shortening **(F)** of left ventricle in both WT male and female mice but not in *nNOS*−/− male and female mice. **(G)** Representative echocardiographic images. **(H,I)** EPO did not change the systolic volume **(H)** and systolic diameter **(I)** in all groups. * <0.05; ** <0.01.

EPO stimulation of NOS activity contributes importantly to EPO multi-organ response including erythroid, cardiovascular, and brain responses (Burger et al., 2009; Mihov et al., 2009; Chen et al., 2010). In mice, overexpression of eNOS attenuated congestive heart failure and improved survival and EPO protection against ischemia-reperfusion injury requires eNOS activity (Burger et al., 2009; Teng et al., 2011a). Neuronal NOS contributes to both EPO-stimulated erythropoiesis and EPO neuroprotective activity in mice (Chen et al., 2010; Lee et al., 2023). However, EPO-stimulated cardiac dysfunction observed here (Figure 1) maybe related to nNOS activity, as suggested by the protective effect of nNOS knockout in mouse models of diastolic dysfunction (Yoon et al., 2021). Therefore, to investigate if the EPO stimulated cardiac dysfunction is related to nNOS activity, we used *nNOS*−/− male and female mice. Metabolic and cardiac responses of male and female WT and *nNOS*−/− mice treated with EPO (3,000 units/kg three times/week) or PBS for 3 weeks on a high-fat diet were determined. Male *nNOS*−/− mice showed less body weight and body fat composition compared to male WT mice, but body weight and fat mass were not significantly different between female *nNOS*−/− and WT mice (Figures 3A, B; Supplementary Figures S2A, B). Mice treated with the NOS inhibitor (N-nitro-L-arginine methyl ester) were reported to be protected against body weight gain on a high-fat diet (Tsuchiya et al., 2007; Sansbury and Hill, 2014). Since targeted deletion of eNOS increases susceptibility to diet-induced obesity (Nakata et al., 2008) and mice with targeted deletion of iNOS on high-fat diet gain weight analogous to WT mice but without an increase in insulin resistance (Perreault and Marette, 2001), this suggests that the loss of nNOS may be protective against diet-induced obesity. The minimal change in body weight and fat mass with high-fat diet feeding in male and female *nNOS−/−* mice is consistent with the anti-obesity effect of NOS inhibition in mice (Sansbury and Hill, 2014) and potentially specific for loss of nNOS. EPO treatment decreased body weight and fat mass accumulation in male WT mice but not in female WT or male and female *nNOS*−/− mice (Figures 3A, B; Supplementary Figures S2A, B), indicating that unlike WT male mice, EPO does not contribute to regulation of fat mass in male or female nNOS−/− mice. EPO treatment improved glucose tolerance in all groups (Supplementary Figures S2C, E), suggesting that EPO-improved glucose tolerance is independent from EPO protection against body fat gain. EPO enhanced insulin tolerance in male and female WT and male *nNOS*−/− mice but did not significantly improve insulin tolerance in female *nNOS*−/− mice (Supplementary Figures S2D, F). EPO increased hematocrit in all groups, male and female WT and *nNOS*−/− mice, and hematocrit tended to be lower in nNOS−/− mice compared with WT mice, without or with EPO treatment (Supplementary Figures S2G, H) (Lee et al., 2023).

### 3.5 EPO treatment did not stimulate cardiac dysfunction in *nNOS*−/− mice

The cardiac function of *nNOS*−/− mice with or without EPO treatment was determined by echocardiography. Measurements in *nNOS*−/− mice showed normal cardiac function.

The PA peak velocity for nNOS−/− mice was similar to WT mice (Figure 3C) and not significantly different from ΔEPORE mice (Figure 1C; Supplementary Figure S3A). Assessment of the LVOT peak velocity, ejection fraction, and fractional shortening in nNOS−/− mice was comparable to WT mice (Figures 3D–G) in contrast to the reduced levels observed for ΔEPORE mice (Figures 1D–F; Supplementary Figures S3B–D). The systolic volume and systolic diameter for nNOS−/− mice were also analogous to WT mice (Figures 3H, I). EPO treatment reduced the PA peak velocity of flow in male and female WT and *nNOS*−/− mice (Figure 3C), in contrast to the PA peak velocity of flow for ΔEPORE mice that was not changed with EPO treatment (Figure 1C; Supplementary Figure S3A). This indicates that modulation of the PA peak velocity by EPO is independent of EPO-stimulated erythropoiesis and requires non-erythroid EPOR expression but does not require nNOS activity. Interestingly, EPO treatment did not change the LVOT peak velocity, ejection fraction, and fractional shortening in male and female *nNOS*−/− mice, while WT male and female mice showed EPO-induced cardiac dysfunction (Figures 3C–F). These data suggest that endogenous nNOS activity contributes critically to EPO-stimulated cardiac modulation and that the loss of nNOS may be protective for decreased heart function with chronic EPO treatment. EPO did not change the systolic volume and diameter in all groups (Figures 3H, I).

### 3.6 EPO treatment did not change heart failure-associated genes in *nNOS*−/− mice

**FIGURE 4:**
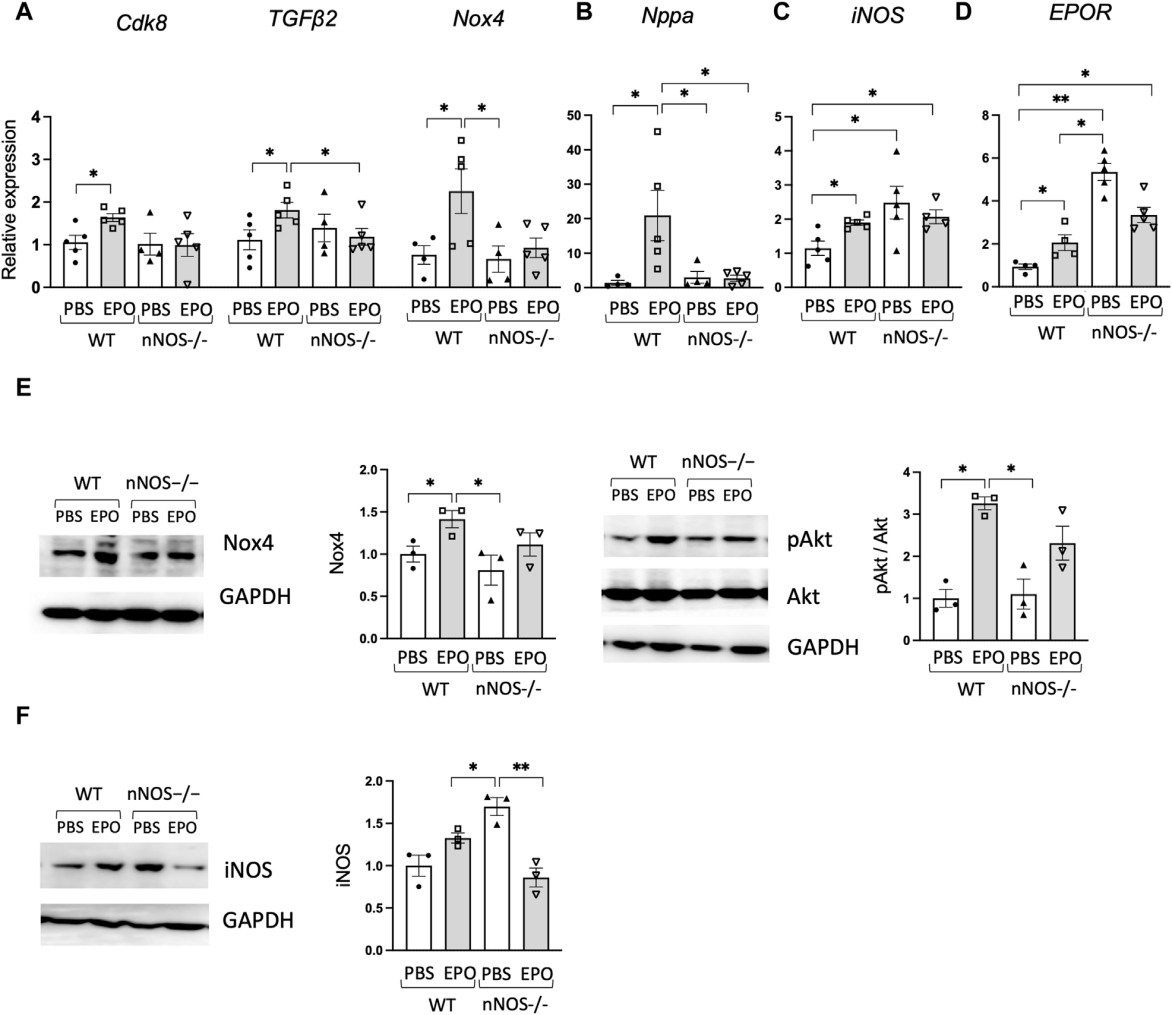
EPO did not change heart failure-associated genes in *nNOS*−/− mice. **(A,B)** EPO enhanced expression of heart failure-associated genes **(A)** and *Nppa* as the hypertrophic cardiomyopathy-related gene **(B)** in WT male mouse cardiac tissue but not in *nNOS*−/− male mouse cardiac tissue (*n* = 4–5). **(C,D)** *iNOS* **(C)** and *EPOR* **(D)** gene expression in WT and *nNOS*−/− male mice with EPO or PBS treatment (*n* = 4–5). **(E)** EPO increased Nox4 and pAkt in WT male mice but not in *nNOS*−/− male mice (*n* = 3). **(F)** Increased iNOS protein was shown in *nNOS*−/− mice compared to WT mice, and EPO decreased iNOS in *nNOS*−/− mice (*n* = 3). * <0.05; ** <0.01.

Loss of EPOR in the non-erythroid tissue of ΔEPORE mice resulted in decreased ejection fraction and fractional shortening (Figure 1) and increased expression of heart failure-associated genes (Figure 2). In contrast, the loss of nNOS did not significantly affect ejection fraction and fractional shortening of nNOS−/− mice (Figure 3) or expression of heart failure-associated genes. In *nNOS*−/− mice, the expression levels of heart failure-associated genes *Cdk8, TGFβ2*, and *NOX4* and hypertrophic cardiomyopathy-related gene *Nppa* were analogous to levels in WT mice (Figures 4A, B). However, while EPO treatment increased expression of these heart failure-associated genes in WT mice, EPO treatment did not change expression of heart failure-associated genes *Cdk8, TGFβ2*, and *NOX4* and hypertrophic cardiomyopathy-related genes *Nppa* in *nNOS*−/− mice (Figures 4A, B). This suggests that the loss of endogenous nNOS activity interferes with EPO-induced upregulation of cardiac function-related genes, which leads to protection against EPO-induced cardiac dysfunction. Expression of Nox4 protein expression that was enhanced by EPO treatment in WT male mice was not significantly different in *nNOS*−/− male mice with EPO treatment, in agreement with corresponding Nox4 gene expression (Figures 4A, E, left). Although EPOR expression was increased in *nNOS−/−* mice (Figure 4D), EPO treatment did not significantly affect heart function in *nNOS−/−* mice (Figure 3). PI3K/Akt signaling participates in myocardial injury, and modulation of PI3K/Akt can protect against ischemia-reperfusion injury or heart failure (Ghafouri-Fard et al., 2022). EPO cardioprotective activity in animal models of ischemia-reperfusion injury in heart was associated with Akt activation (Parsa et al., 2003; Cai and Semenza, 2004; Kobayashi et al., 2008). We found that EPO treatment increased activation of the Akt signaling pathway indicated by increased Akt phosphorylation in WT mice but did not significantly increase phosphorylated Akt relative to total Akt in nNOS−/− mice (Figure 4E, right), suggesting that pAkt signaling might be critical for EPO response in cardiac function mediated by endogenous nNOS. Surprisingly, increased iNOS expression and protein was observed in *nNOS*−/− mice compared to WT mice (Figures 4C, F), and EPO treatment decreased iNOS protein in *nNOS*−/− mice (Figure 4F), suggesting modulation of inflammation in *nNOS−/−* mice without affecting the cardiac function.

## 4 Discussion

EPO functions primarily to regulate the daily production of approximately 200 billion red blood cells to transport oxygen from the lungs to the tissues (Bhoopalan et al., 2020). Although EPOR expression is the highest on differentiating erythroid progenitor cells, EPOR expression extends to non-erythroid cells (Suresh et al., 2019b). Animal models provide evidence of non-hematopoietic response to EPO that includes improved metabolic activity and potential protection to injury in brain, heart, and skeletal muscle (Katz et al., 2010; Ueda et al., 2010; Teng et al., 2011b; Jia et al., 2012; Singh et al., 2023). Although both male and female WT and ΔEPORE mice showed similar increases in hematocrit and improvement in glucose tolerance and insulin tolerance with EPO treatment, male WT mice on high-fat diet feeding exhibited the greatest reduction in body weight and fat mass with EPO treatment (Figure 1; Supplementary Figure S1) (Zhang et al., 2017). The minimal change in body weight and fat mass in ΔEPORE mice with EPO treatment indicates that EPO regulation of fat mass is mediated via *EPOR* expression in non-erythroid tissue (Teng et al., 2011b). Little or no change in body weight and fat mass was apparent in WT female mice on a high-fat diet treated with EPO (Supplementary Figure S1) due to the protective effect of estrogen against diet-induced obesity mediated via *estrogen receptor α* (Zhang et al., 2017; Lee et al., 2021). Similarly, male and female *nNOS−/−* mice on a high-fat diet showed little change in body weight and fat mass with EPO treatment (Figure 3; Supplementary Figure S2), consistent with the anti-obesity effect of NOS inhibition (Sansbury and Hill, 2014) that may be specific to the loss of nNOS activity. A possible direct cardiovascular effect of EPO beyond increasing red blood cell production was suggested by the proliferative and chemo-attractive response to EPO stimulation of endothelial cells that express endogenous *EPOR* (Anagnostou et al., 1990; Anagnostou et al., 1994). A potential association between elevated EPO and cardioprotection is suggested by increased EPO production and erythropoiesis associated with treatment with sodium-glucose cotransporter 2 (SGLT2) inhibitors for metabolic disorders that are associated with a reduced risk of heart failure events (Packer, 2020; Packer et al., 2020; Packer, 2021). A specific role for endogenous EPO in cardiovascular development was provided by mice lacking *EPO* or *EPOR* that exhibit developmental angiogenic and cardiac defects including ventricular hyperplasia prior to death *in utero* due to severe anemia (Wu et al., 1999; Yu et al., 2001; Kertesz et al., 2004). Here, we found that endogenous EPO contributes to maintaining the heart function. Without EPO treatment, ΔEPORE mice exhibited a decreased LVOT peak velocity, ejection fraction, and fractional shortening, and increased systolic volume and systolic diameter compared with WT mice (Figure 1). These changes in heart function were accompanied by increased expression in heart failure-associated genes, hypertrophic cardiomyopathy-related genes, and sarcomeric genes, and activation of ERK signaling in ΔEPORE mice (Figure 2).

Unlike the protective effect of acute EPO administration in animal models of cardiac ischemia-reperfusion injury (Burger et al., 2006; Teng et al., 2011a), mice exposed to chronic high-dose EPO exhibit adverse effects on bone and heart health (Wagner et al., 2001; Hiram-Bab et al., 2015; Suresh et al., 2019a). Here, we found that 3 weeks of EPO treatment compromised heart function with a decreased PA peak velocity, LVOT peak velocity, ejection fraction, and fractional shortening in male and female WT mice but not in ΔEPORE mice (Figure 1), indicating that modulation of heart function with EPO treatment is due to a non-erythroid response to EPO and independent of EPO-stimulated erythropoietic response. Surprisingly, we found that nNOS activity was required for EPO modulation of heart function and decrease in LVOT peak velocity, ejection fraction, or fractional shortening as these changes were not significantly altered with EPO treatment in *nNOS*−/− mice (Figure 3). LVOT peak velocity, ejection fraction, and fractional shortening in *nNOS−/−* mice were comparable to WT mice and were not affected by EPO treatment. Unlike WT mice, EPO treatment did not increase expression of heart failure-associated gene expression in *nNOS*−/− mice (Figure 4). With EPO treatment, *nNOS−/−* mice showed improved glucose tolerance and insulin tolerance with EPO treatment but no change in body weight or fat mass (Figure 3; Supplementary Figure S2). Although the loss of *nNOS* expression appears to be protective in modulation of heart function by EPO treatment, *nNOS−/−* mice exhibit a tendency toward a blunted EPO-stimulated erythropoietic response (Supplementary Figure S2) (Lee et al., 2023). The reduction in heart function with EPO treatment (3 weeks) in WT mice is consistent with the cardiac dysfunction of transgenic mice with chronic expression of high *EPO* (Wagner et al., 2001). However, the increased hematocrit with EPO treatment in ΔEPORE mice and *nNOS*−/− mice indicates that EPO-stimulated erythropoiesis does not account for the adverse effect of elevated EPO on heart function. Rather, the resultant change in heart function with prolonged EPO treatment is a non-erythroid activity of EPO that requires both *nNOS* expression and EPOR in non-erythroid tissue. This activity of EPO contrasts the cardioprotective effect of acute EPO treatment in animal models that requires *eNOS* expression (Burger et al., 2006; Teng et al., 2011a).

Studies presented here exemplify the link between EPO and NOS activity. EPO regulation of NO production provides an additional mechanism for EPO to improve the oxygen delivery beyond erythropoiesis by regulating vascular tone. EPO activates eNOS and NO production in endothelial cells, especially at reduced oxygen, and mice expressing high transgenic EPO with a hematocrit of 80% exhibit high eNOS activity and NO production to regulate blood pressure (Ruschitzka et al., 2000; Beleslin-Cokic et al., 2004; Beleslin-Cokic et al., 2011). In these mice, treatment with the NOS inhibitor (N-nitro-L-arginine methyl ester) decreased eNOS and NO production, increased vasoconstriction and hypertension, and reduced mouse survival (Quaschning et al., 2003). In animal models, EPO exhibited cardioprotective activity in ischemia-reperfusion injury of isolated adult hearts (Cai et al., 2003; Parsa et al., 2003), and *in vivo*, in myocardial infarction, long-term EPO treatment induced neovascularization (Cai and Semenza, 2004; van der Meer et al., 2005; Westenbrink et al., 2010). Activation of eNOS was required for EPO cardioprotective effects that included increased coronary endothelial production of NO (Mihov et al., 2009; Teng et al., 2011a). A link between EPO and nNOS activity is suggested by EPO response in the brain. EPO is associated with NO-induced axonal protection and contributes to the NO-dependent ventilatory response to hypoxia in mice (Keswani et al., 2011; Voituron et al., 2014). NO stimulated neural cell expression of EPOR, particularly at reduced oxygen (Chen et al., 2010). During mouse development and prior to death *in utero* due to severe anemia, mice that lack *EPOR* exhibit reduced neural progenitor cells, hypoxia sensitivity, and increased brain apoptosis (Liu et al., 1997; Yu et al., 2002). EPOR and EPO expression in neurons and neural cells contribute to neuroprotection, hypoxia preconditioning, and neurogenesis in animal models (Sadamoto et al., 1998; Sakanaka et al., 1998; Bernaudin et al., 1999; Iwai et al., 2007). Translating EPO neuroprotective activity from animal models to the clinic has been difficult. EPO showed potential for treatment of ischemic stroke in a Phase I clinical trial, but the beneficial effect of EPO administration in ischemic stroke was not realized in an expanded Phase II/III clinical trial (Ehrenreich et al., 2002; Ehrenreich et al., 2009).

Although EPO has been used for more than three decades to treat anemia in patients with chronic kidney disease, the cardioprotective effects of EPO suggested in animal models have been difficult to demonstrate in humans. A rat model of chronic kidney diseases indicated that the myocardial protective activity of erythropoiesis-stimulating agents is lost at the increased dose required to normalize hemoglobin levels (Miura et al., 2022). Cardiac ischemia-reperfusion injury in rats also showed that EPO protective activity on mitochondrial dysfunction depended on the timing of EPO administration (Benjanuwattra et al., 2022). A mouse model of myocardial ischemia-reperfusion injury provided evidence that SOCS3 activity inhibited the protective effect of myocardial ischemic preconditioning mediated by increased kidney EPO production (Nohara et al., 2021). In mice, EPO increased hypertension *in vivo* and contractility in mouse aortic smooth muscle cells *in vitro* (Sun et al., 2021). In patients with acute ST-segment elevation myocardial infarction with successful percutaneous coronary intervention as a reperfusion strategy, a single dose of EPO treatment did not reduce the infarct size and raised concerns about increase rates of adverse cardiovascular events in older patients (Najjar et al., 2011). In contrast, a small pilot study using low-dose EPO treatment suggested improvement of the left ventricular function, although a multicenter study found no improvement in the cardiac function and no increase in adverse events (Minamino et al., 2012; Minamino et al., 2018). However, exogenous EPO treatment to renal failure patients was associated with an increased risk of hypertension and cardiovascular events (Besarab et al., 1998; Singh et al., 2006), and higher levels of endogenous EPO in healthy individuals were associated with higher incidence of heart failure (Garimella et al., 2016; Sun et al., 2021).

NOS contributes importantly to maintaining cardiac homeostasis. Mice lacking *eNOS*, *nNOS*, and *iNOS* exhibit significant left ventricular hypertrophy at 5 months, which is observed to a lesser extent in *eNOS*−/− mice but not in *nNOS*−/− or *iNOS*−/− mice (Shibata et al., 2010). Both cardioprotective and detrimental activities of nNOS have been observed using various mouse models of cardiac function. The cardioprotective effect of β3-adrenergic receptor activation on modulating cardiovascular function, left ventricular dilation, and heart failure is associated with NO production and nNOS activation (Niu et al., 2012). EPO treatment was observed to increase nNOS expression in ventricular myocytes, and EPO was protective in a mouse model of myocardial ischemia-reperfusion injury from CsCl-induced ventricular arrhythmia via nNOS activity (Burger et al., 2009). In contrast, in a mouse model of diastolic dysfunction, impaired diastolic function and exercise tolerance were linked to nNOS activity, and mice maintained a normal diastolic function with inhibition or knockout of *nNOS* attributed to changes in S-nitrosylation of proteins such as histone deacetylase 2 (Yoon et al., 2021). In a “two-hit” mouse model for heart failure with preserved ejection fraction, the combination of a high-fat diet and inhibition of NOS (N-nitro-L-arginine methyl ester) reduced unfolded protein response effector X-box-binding protein 1 in the heart and increased iNOS activity that were critical for the resultant diastolic dysfunction (Schiattarella et al., 2019). In this mouse model of heart failure with preserved ejection fraction, specific inhibition of iNOS improved ventricular relaxation. The cardioprotective activity of cardiac-specific overexpression of plasma membrane Ca+2-ATPase 4 in mice is associated with decreased total NOS in the heart and cardiac nNOS activity, and this activity of cardiac-specific overexpression of plasma membrane Ca+2-ATPase 4 is lost with increased NO availability (Sadi et al., 2018).

The apparent cardioprotective effect in *nNOS*−/− mice to extended high-dose EPO treatment to increase hematocrit is in marked contrast to the decrease erythropoietic response of *nNOS*−/− mice to EPO stimulation (Lee et al., 2023). NO and regulated protein S-nitrosylation contribute importantly to regenerating hematopoietic stem cells (Yi et al., 2021). The post-translational modification of S-nitrosylation of proteins by nNOS including histone deacetylase 2 that contributes to compromised heart function in mice may affect hematopoiesis and differentiation along the erythroid lineage to promote effective erythropoietic response to EPO stimulation. Although the extent of protein S-nitrosylation and candidate proteins that may be modified during erythropoiesis is not known, there may be common targets for the EPO-stimulated post-translational modification by nNOS and S-nitrosylation that regulate erythropoiesis and heart function.

These findings reveal the potential for high-dose EPO treatment to compromise heart function, independent of increased hematocrit. In contrast to earlier observations in animal models that demonstrated the cardioprotective effect of single-dose EPO treatment in heart ischemia-reperfusion injury, extended high-dose EPO administration to increase hematocrit negatively impacts on heart function in mice. Both these protective and adverse effects of EPO administration are independent of EPO-stimulated erythropoiesis and require NOS activity, and nNOS−/− mice are protected from the adverse effects of EPO on the heart health.

## Scope

Erythropoietin (EPO) is the primary regulator of erythropoiesis, although EPOR expression extends beyond erythroid tissue, providing for non-hematopoietic EPO response. In animal models, nitric oxide (NO) synthase (NOS) and NO production contribute to EPO activity including erythropoiesis and neuro- and cardioprotection. However, high-dose EPO treatment in patients with chronic kidney disease was linked to an increased risk of serious cardiovascular events and mortality. We found that while heart function in mice is protected by endogenous EPO, heart function is impaired by prolonged EPO treatment to increase hematocrit. Impaired heart function by EPO treatment is independent of EPO-stimulated erythropoiesis and requires EPOR in non-erythroid tissue. Increases in expression of heart failure-associated genes were consistent with decreased heart function. Although nNOS−/− mice show increased hematocrit with EPO treatment, albeit with a trend toward lower hematocrit compared with wild-type mice, nNOS−/− mice are protected from the adverse effect of EPO treatment on heart function. These findings reveal the potential for high-dose EPO treatment to compromise heart function, independent of increased hematocrit, and the contribution of NOS activity to both the beneficial and adverse effects of EPO and raise the potential for a role for protein S-nitrosylation in EPO activity.

## Data Availability

The original contributions presented in the study are included in the article/Supplementary Material; further inquiries can be directed to the corresponding author.
