# Supplementary material for: Neuronal nitric oxide synthase required for erythropoietin modulation of heart function in mice
Source: Front Physiol. 2024 Apr 2;15:1338476. doi: 10.3389/fphys.2024.1338476 (PMC11019009; doi:10.3389/fphys.2024.1338476)
Supplement: Supplementary file 1 [file Image5.pdf]

Supplementary Figure S5. Images for Western blotting for WT and *nNOS-/-* mice

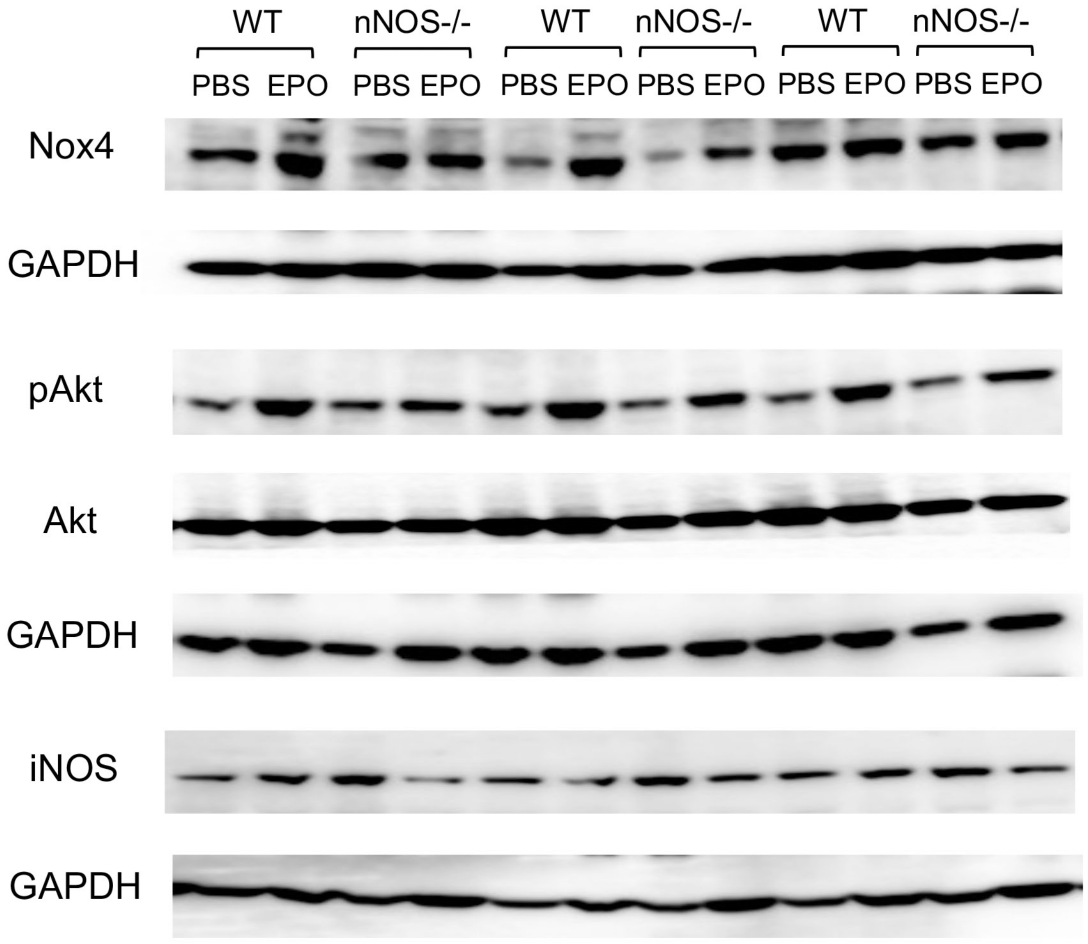

Supplementary Figure S5. Images for Western blotting for WT and *nNOS*-/- mice
Original images from Western blotting for NOX4, GAPDH, pERK, ERK and GAPDH for heart tissues from WT and *nNOS-/-* mice.
