# Supplementary material for: Neuronal nitric oxide synthase required for erythropoietin modulation of heart function in mice
Source: Front Physiol. 2024 Apr 2;15:1338476. doi: 10.3389/fphys.2024.1338476 (PMC11019009; doi:10.3389/fphys.2024.1338476)
Supplement: Supplementary file 2 [file Table1.docx]

Supplemental Table S1. Primers sequences for qPCR

| Primers | Forward | Reverse |
| --- | --- | --- |
| *Cdk8* | GACTATCAGCGTTCCAATCCAC | TAGCTGAGTATCCCATGCTGC |
| *TGFβ2* | CCACCTCCCCT CCGAAAA | AGACATCAAAGCGGACGATTCT |
| *Nox4* | GATCACAGAAGGTCCCTAGCA | GTTGAGGGCATTCACCAAGT |
| *S100A* | ccatggagaccctcatcaat | ttgaagtccacttccccatc |
| *SERCA2a* | CGGTGCCTTTGTTGTCTCCA | ACCTGACTTTCGTCGGCTGTGT |
| *Acta1* | CGACATCAGGAAGGACCTGTATGCC | AGCCTCGTCGTACTCCTGCTTGG |
| *Myh7* | TTCCTTACTTGCTACCCTC | CTTCTCAGACTTCCGCAG |
| *Nppa* | CCAGGCCATATTGGAGCAAA | GAAGCTGTTGCAGCCTAGTC |
| *Nppb* | GCTGCTTTGGGCACAAGATAG | GCAGCCAGGAGGTCTTCCTA |
| *Tcap* | CCGGAAGAGGGATGCTCCT | CTGGTACGGCAGCTGGTATT |
| *Myom2* | CTGGCTCTTCTCCTTTGGGAAC | CCAGGAGGTACCTGGTTTGGA |
| *Myoz2* | GCCCAAGTGCCGTCATATTC | CATGCCGTCAACATCATGTCC |
| *EPOR* | GCTCCGGGATGGACTTCA | GAGCCTGGTGCAGGCTACAT |
| *iNOS* | CAGCTGGGCTGTACAAACCTT | CATTGGAAGTGAAGCGTTTCG |
| *RPL13a* | GCTTCTTCTTCCGATAGTGCATC | AGCCTACCAGAAAGTTTGCTTAC |
