# Supplementary material for: Neuronal nitric oxide synthase required for erythropoietin modulation of heart function in mice
Source: Front Physiol. 2024 Apr 2;15:1338476. doi: 10.3389/fphys.2024.1338476 (PMC11019009; doi:10.3389/fphys.2024.1338476)
Supplement: Supplementary file 3 [file Image4.pdf]

Supplementary Figure S4. Images for Western blotting for WT and ΔEPORE mice

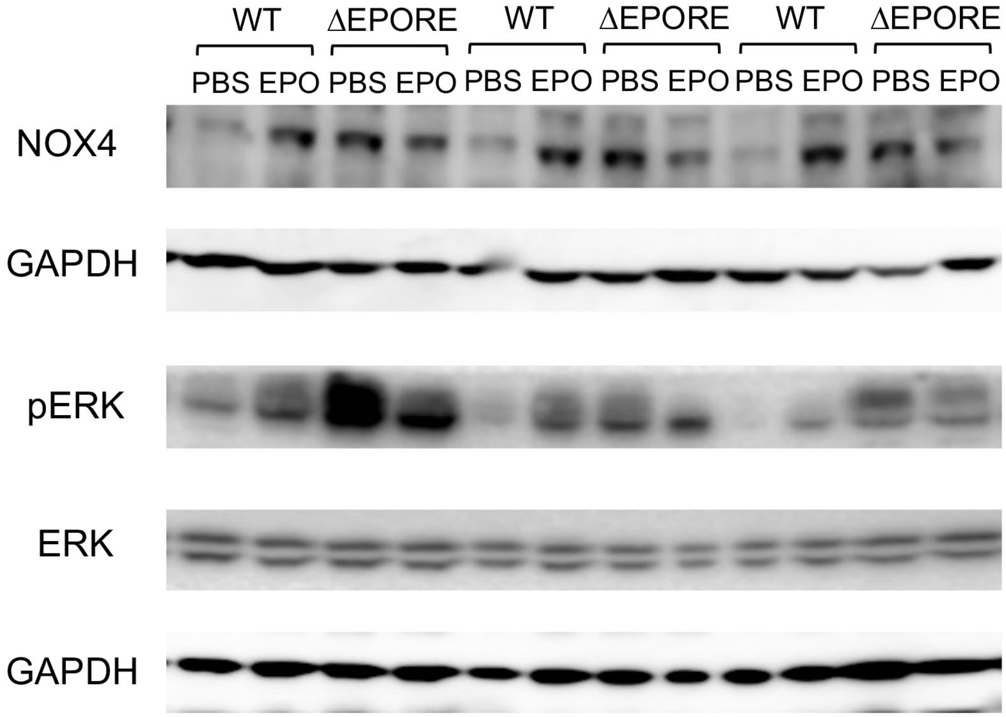

Supplementary Figure S4. Images for Western blotting for WT and ΔEPORE mice
Original images from Western blotting for NOX4, GAPDH, pERK, ERK and GAPDH for heart tissues from WT and ΔEPORE mice.
