# Supplementary material for: Neuronal nitric oxide synthase required for erythropoietin modulation of heart function in mice
Source: Front Physiol. 2024 Apr 2;15:1338476. doi: 10.3389/fphys.2024.1338476 (PMC11019009; doi:10.3389/fphys.2024.1338476)
Supplement: Supplementary file 4 [file Image2.pdf]

Supplementary Fig S2. Fat mass and hematocrit changes in nNOS-/- mice with EPO treatments

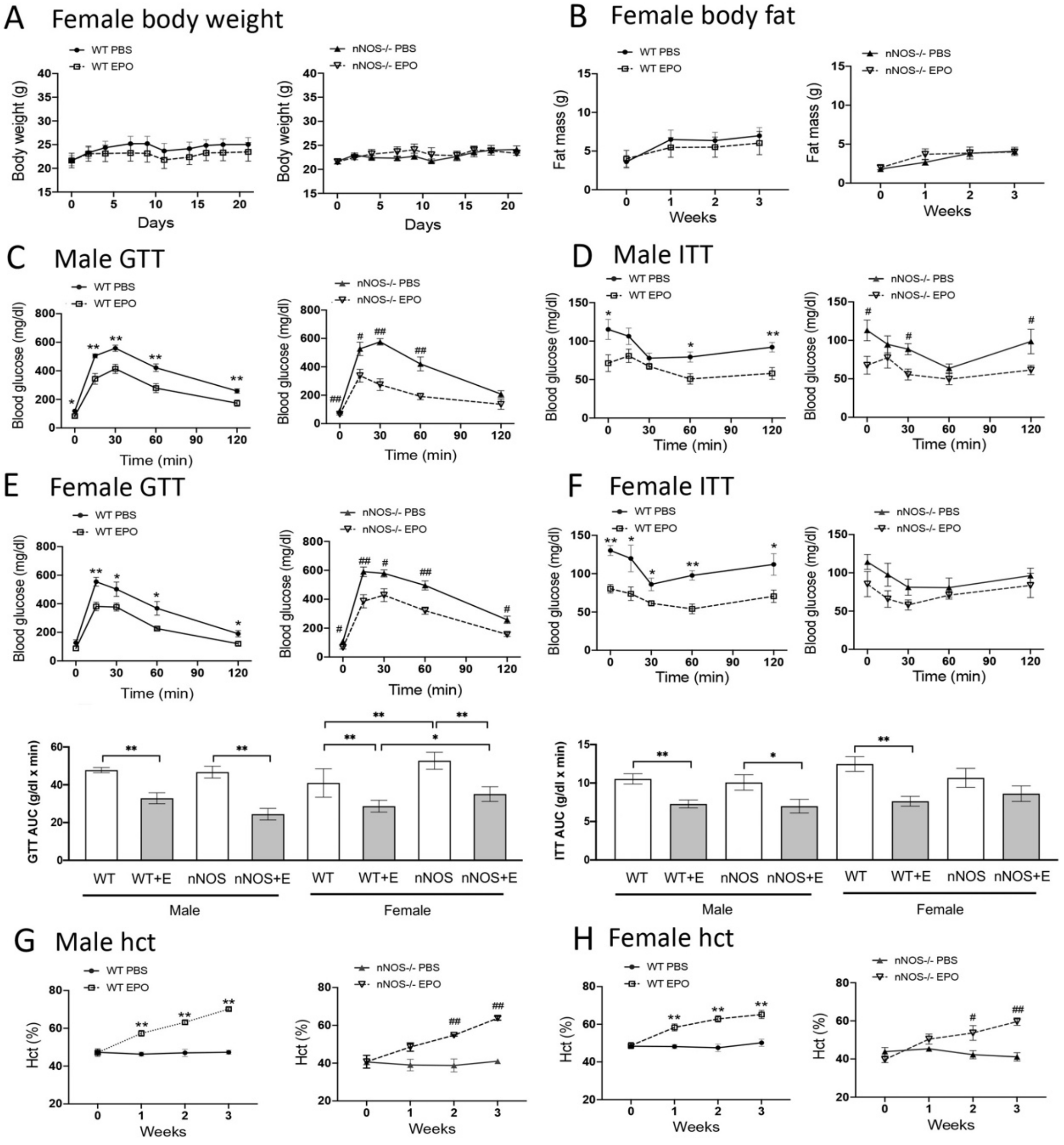

Supplementary Figure S2. Fat mass and hematocrit changes in *nNOS*-/- mice with EPO treatment

A-B. Body weight (A) and fat mass accumulation (B) with PBS or EPO treatment with high fat diet in WT female or *nNOS*-/- mice. C-F. EPO improved glucose tolerance in all groups (C and E) and insulin tolerance (D and F) in WT male and female and *nNOS*-/- male mice, but not in *nNOS*-/- female mice. G-H. EPO treatment increased hematocrit in all groups, WT and *nNOS*-/- male mice (G) and WT and *nNOS*-/- female mice (H). n=4-7, *, ** mean PBS vs EPO in WT mice, #, ## mean PBS vs EPO in *nNOS*-/- mice. *, # <0.05, **, ## <0.01
