# Supplementary material for: Neuronal nitric oxide synthase required for erythropoietin modulation of heart function in mice
Source: Front Physiol. 2024 Apr 2;15:1338476. doi: 10.3389/fphys.2024.1338476 (PMC11019009; doi:10.3389/fphys.2024.1338476)
Supplement: Supplementary file 5 [file Image3.pdf]

Supplementary Figure s3. Comparison of heart function in ΔEPORE and *nNOS*-/- mice

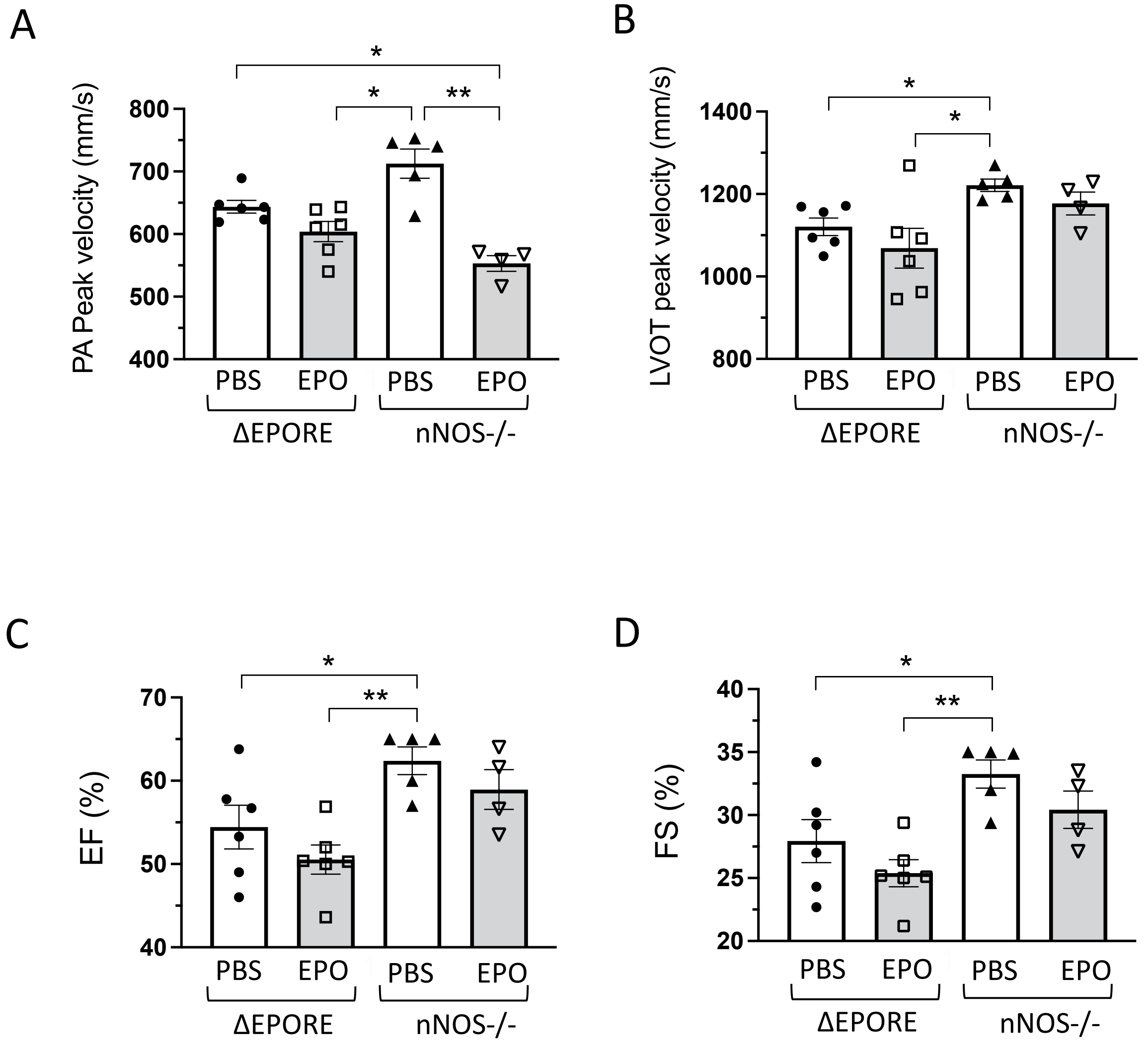

Supplementary Figure s3. Comparison of heart function in ΔEPORE and *nNOS*-/- mice
A-D. All male mice were place on high fat diet concomitant with EPO and PBS treatment for 3 weeks (See Figure 1 and Figure 3). A. EPO treatment with high fat diet significantly reduced PA peak velocity only in *nNOS*-/- mice. B. *nNOS*-/- showed increased LVOT peak velocity compared to ΔEPORE. C-D. ΔEPORE mice had lower level of ejection fraction (C) and fractional shortening (D) compared to *nNOS*-/- mice. * <0.05, ** <0.01. All data were analyzed by a one-way ANOVA Kruskal-Wallis test and a subsequent Dunn's test.
