# Supplementary material for: Neuronal nitric oxide synthase required for erythropoietin modulation of heart function in mice
Source: Front Physiol. 2024 Apr 2;15:1338476. doi: 10.3389/fphys.2024.1338476 (PMC11019009; doi:10.3389/fphys.2024.1338476)
Supplement: Supplementary file 6 [file Image1.pdf]

Supplementary Figure S1. Hematocrit, fat mass, and insulin resistance changes in WT and ∆EPORE mice with EPO treatment

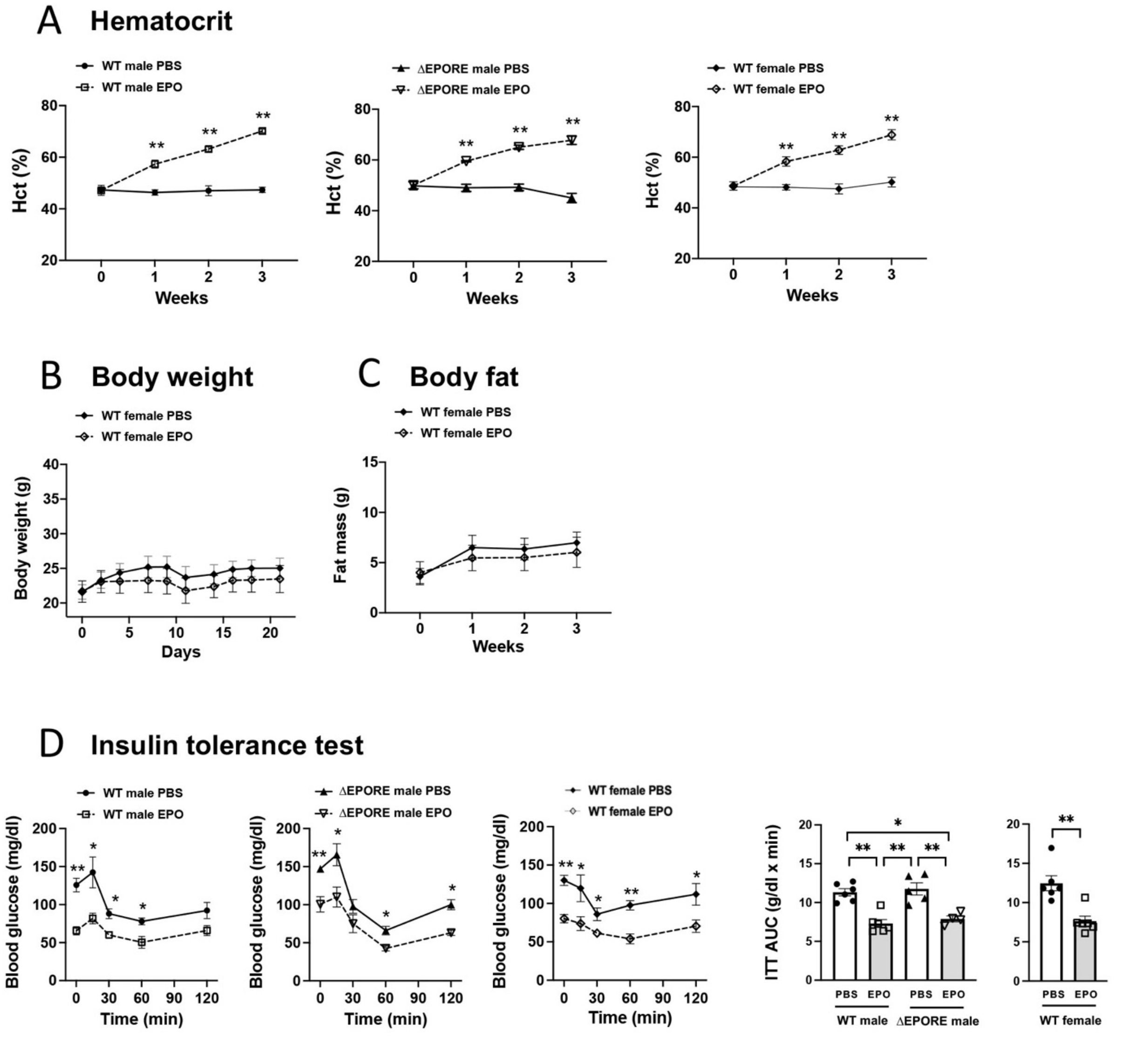

Supplementary Figure S1. Hematocrit, fat mass, and insulin resistance changes in WT and ∆EPORE mice with EPO treatment. A. EPO treatment increased hematocrit similarly in all groups, male and female WT mice, and ∆EPORE male mice. B-C. Body weight (B) and fat mass accumulation (C) in WT female and ΔEPORE male mice. D. EPO improved insulin tolerance in all groups, WT male, ∆EPORE male, and WT female mice. Area under the curve for the insulin tolerance test (ITT AUC) is also shown. n=4-7, * <0.05, ** <0.01.
